# Small pigmented eukaryote assemblages of the western tropical North Atlantic around the Amazon River plume during spring discharge

**DOI:** 10.1038/s41598-021-95676-2

**Published:** 2021-08-10

**Authors:** Sophie Charvet, Eunsoo Kim, Ajit Subramaniam, Joseph Montoya, Solange Duhamel

**Affiliations:** 1 Lamont-Doherty Earth Observatory, Columbia University, New York, NY USA; 2 American Museum of Natural History, New York, NY USA; 3 Georgia Institute of Technology, Atlanta, GA USA; 4 Present Address: University of Arizona, Tucson, AZ USA

**Keywords:** Biodiversity, Marine biology, Microbial ecology

## Abstract

Small pigmented eukaryotes (⩽ 5 µm) are an important, but overlooked component of global marine phytoplankton. The Amazon River plume delivers nutrients into the oligotrophic western tropical North Atlantic, shades the deeper waters, and drives the structure of microphytoplankton (> 20 µm) communities. For small pigmented eukaryotes, however, diversity and distribution in the region remain unknown, despite their significant contribution to open ocean primary production and other biogeochemical processes. To investigate how habitats created by the Amazon river plume shape small pigmented eukaryote communities, we used high-throughput sequencing of the 18S ribosomal RNA genes from up to five distinct small pigmented eukaryote cell populations, identified and sorted by flow cytometry. Small pigmented eukaryotes dominated small phytoplankton biomass across all habitat types, but the population abundances varied among stations resulting in a random distribution. Small pigmented eukaryote communities were consistently dominated by Chloropicophyceae (0.8–2 µm) and Bacillariophyceae (0.8–3.5 µm), accompanied by MOCH-5 at the surface or by Dinophyceae at the chlorophyll maximum. Taxonomic composition only displayed differences in the old plume core and at one of the plume margin stations. Such results reflect the dynamic interactions of the plume and offshore oceanic waters and suggest that the resident small pigmented eukaryote diversity was not strongly affected by habitat types at this time of the year.

## Introduction

The Amazon River plume (ARP) results from the massive freshwater discharge of the Amazon River into the western tropical North Atlantic Ocean and at its peak can cover an area greater than 1 million km^1,2^. Density-driven mixing of the buoyant freshwater plume creates various strong local gradients in salinity, inorganic nutrients and turbidity. During peak discharge in the spring, the ARP is pushed northwards by the North Brazil current along the eastern coast of South America, then is carried eastwards later in the year by the North Equatorial Countercurrent^2^. During this northward progression, the plume mixes gradually into the coastal and offshore waters on either side, establishing a diverse range of ecological niches impacting phytoplankton community structure far from the mouth of the river^1,3,4^.

Changes in the phytoplankton community structure are primarily driven by the nutrient gradients along the aging plume^3^. With its load of inorganic nitrogen, phosphorus and silicate, the ARP enhances primary production in the western tropical North Atlantic Ocean^1^. There is a rapid drawdown of dissolved inorganic nitrogen by diatoms (≥ 10 µm) in the young freshwaters of the plume, and the more mature mesohaline waters are characterized by diatom-diazotroph associations^1^. The older sections of the plume are depleted in nitrogen and dominated by cyanobacterial diazotroph *Trichodesmium* colonies^1,4,5^. The progressive shift in dominance of these N_2_-fixing populations promotes the particular co-occurrence of other micro-phytoplankton groups, including diatoms and dinoflagellates, making up to three distinct assemblages^3^. Despite extensive studies of the ARP region, very little has been reported on the small (≤ 5 µm) phytoplankton communities and the focus has only been on picocyanobacteria^3,5,6^.

Among phytoplankton communities, the small-sized pigmented eukaryotes (≤ 5 µm) are ubiquitous throughout the global oceans, reaching peak abundance at the deep chlorophyll maximum^7–9^ or at the base of the surface mixed layer^10^. At these depths, small pigmented eukaryotes sometimes surpass picocyanobacteria in biomass^11,12^ and can even contribute up to half or more of picophytoplankton (typically defined as including *Prochlorococcus*, *Synechococcus* and pigmented eukaryotes ≤ 2–3 µm) primary production^12–15^. Along horizontal gradients, while *Prochlorococcus* dominates in abundance in oligotrophic waters^11^, small pigmented eukaryotes can contribute a higher proportion of the small-sized phytoplankton abundance at the nutrient-rich coastal stations^10,12,16–18^. Similarly, the taxonomic composition of small pigmented eukaryote assemblages varies along oceanic gradients, ranging from a significant contribution of small Chlorophyta in nutrient-rich coastal regions^8,15,19,20^ to the dominance of prymnesiophytes^8,16,21–23^, chrysophytes^21^ or dinoflagellates^13^ in oligotrophic open-ocean waters. Small diatoms of < 2–5 µm can also make up a significant portion of the spring bloom, as observed in the Mediterranean Sea and in global datasets^24^. Additionally, taxonomic variability is observed along latitudinal gradients with a marked increase in eukaryote contribution to small-sized phytoplankton communities, mostly by Chlorophyta, from the North Pacific to the Arctic^25–27^.

River influenced coastal systems are characterized by gradients of salinity, turbidity and nutrients, which affect primary production, as observed for the Rhône River^28^, the Mississippi River^29^ and the Amazon River^30^. Small pigmented eukaryotes seem to be abundant in zones of mixing between the seawater and freshwater which create an optimal trade-off between nutrient concentrations and turbidity^31,32^. However, to our knowledge, there are no studies on how such gradients affect the taxonomic composition of small pigmented eukaryote communities. This work aims to provide a detailed description of the heretofore largely uncharacterized small pigmented eukaryote communities in the western tropical North Atlantic Ocean^33,34^. Considering the multiple gradients that define the ARP, we hypothesized that small pigmented eukaryote communities vary widely across the different habitat types previously described by Weber et al*.*^35^. Given the global tendency of small pigmented eukaryote to thrive at deep chlorophyll maxima, we also hypothesized that their diversity varies vertically within the water column. To test these hypotheses, we used fluorescence-activated cell sorting (FACS) in combination with 18S rRNA gene sequencing for the detailed taxonomic description of distinct small pigmented eukaryote cell populations distinguished by flow cytometry based on cellular characteristics (i.e. flow-cytometric cell populations, hereafter populations^36^).

## Results and discussion

### Habitat types

**Figure 1:**
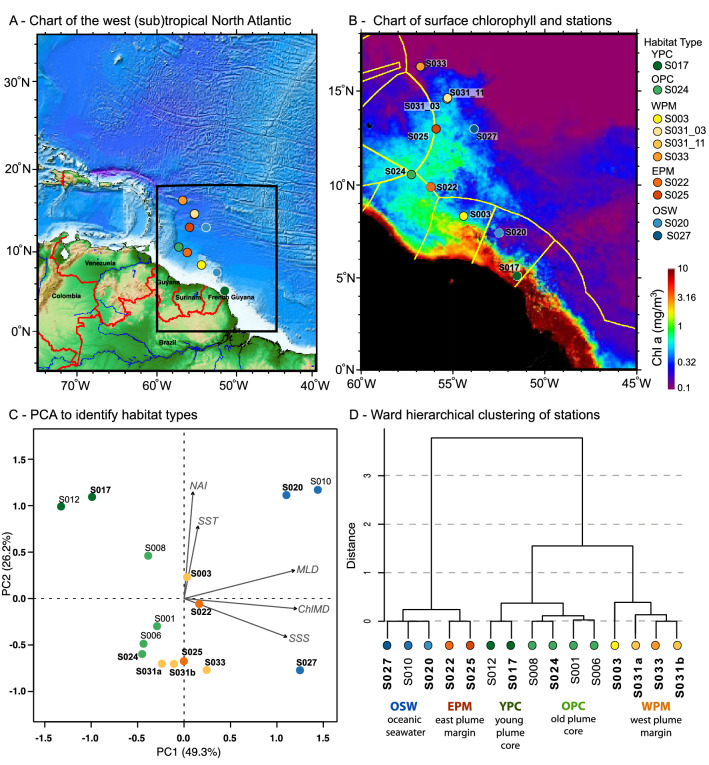
Location of the study (**A**), distribution of sampling stations (**B**) and identification of the habitat types using a principal component analysis (**C**) and Ward’s hierarchical cluster analysis (**D**). The map in B shows the monthly composite surface chlorophyll concentration for May 2018 from satellite observations Reprocessed L4 (ESA-CCI: OCEANCOLOUR_GLO_CHL_L4_REP_OBSERVATIONS_009_093) downloaded from Copernicus Marine Service (https://resources.marine.copernicus.eu). The map was created using the NASA SeaDAS 7.5.3 software with land and exclusive economic zones boundaries (yellow lines) added with gmt v5.4.5 software. Note that all stations from EN614 were used to establish habitat types, but only the 10 stations highlighted in bold and shown on the map were used in this study. SSS, sea surface salinity; SST, sea surface temperature; NAI, nitrogen availability index; MLD, mixed layer depth; ChlMD, chlorophyll maximum depth.

The sampled stations were classified into 5 habitat types (Fig. 1a,b) as described by Weber et al*.*^35^: young plume core (YPC), old plume core (OPC), west plume margin (WPM), east plume margin (EPM) and oceanic seawater (OSW). Each habitat was characterized by a unique combination of sea surface salinity, sea surface temperature, nitrate availability index, mixed layer depth and chlorophyll maximum depth^35^. Geographically, the different habitats were unevenly distributed along the transect (Fig. 1c), illustrating the dynamic and patchy nature of the ARP. At each station, the temperature and salinity profiles confirmed the stratification of the water column. Maximum Brunt–Väisälä buoyancy frequency was high (3–15 × 10^–3^ s^−1^) and close to the surface in the plume core (YPC and OPC), restricting turbulent mixing between the plume waters and the underlying ocean waters. The plume margin stations (WPM and EPM) showed deeper and more muted (1–2 × 10^–3^ s^−1^) maximum buoyancy frequency peaks while OSW stations exhibited turbulent mixing from the surface to ~ 100 m (Supplementary Fig. S1). Fluorescence profiles provided guidance to sample within the chlorophyll maximum (Supplementary Fig. S1). In the plume core, the chlorophyll peak was located above the halocline. At plume margin stations, multiple chlorophyll maxima were detected at the halocline or just below, while the oceanic seawater stations did not have haloclines, and chlorophyll peaks were far below the surface (deeper than 50 m). Surface samples from the core plume stations corresponded to high temperature-low salinity waters, with low density. These plume waters mixed with coastal waters at the surface of plume margin stations, but this was not the case at OSW stations (Supplementary Fig. S2).

### Small-sized pigmented eukaryote populations, size, abundance and biomass:

**Figure 2:**
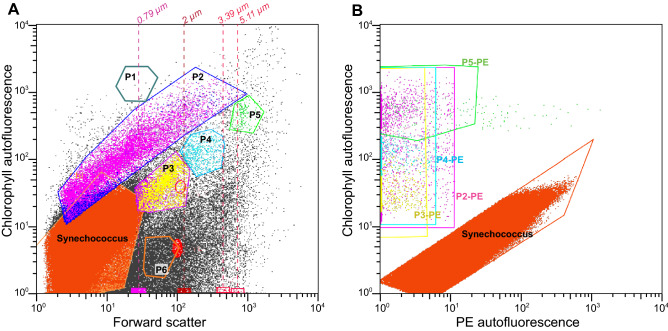
Example of a cytogram illustrating the gates used for small pigmented eukaryote population counts and sorting. Populations were first discriminated based on their position in the chlorophyll vs forward scatter cytogram (**A**, all events represented) and then redefined in the chlorophyll vs phycoerythrine (PE) autofluorescence cytogram (**B**, only events gated in A represented). In the later, we avoided *Synechococcus* overlapping with small pigmented eukaryote populations in A and cells exhibiting high PE fluorescence among the populations from cytogram B. Note that all 6 populations were never found present in the same sample. In particular, the sample represented here (S003 surface) did not contain P1 or P6, but the gates are represented nonetheless in panel A to provide an illustration for these populations. Note that the gating had to be adjusted between samples but the relative positions stayed similar to those illustrated here. The positions of standard size-calibrated non-fluorescent beads (dashed lines) along the x-axis were used to determine the size range of each gated population in cytogram A. Red ellipses mark the position of yellow-green reference beads of 1 and 2 µm (1-YG and 2-YG, respectively) used to maintain instrument alignment, although the bead clusters are not apparent in the sample since they were run separately (for details see “Methods”).

Overall, the small pigmented eukaryote communities were composed of a variable combination of 3 to 4 populations per sample, with a total of 6 different populations (named P1, P2, P3, P4, P5 and P6) among all samples, identified by flow cytometry according to cell size range and pigment content (Fig. 2). Based on relative estimates from flow cytometry calibrations using beads of known sizes, most populations belonged to the picoplankton (≤ 2–3 µm). Cells in P1 were approx. 0.8 µm. P2 was a very diverse cluster resulting in a size range from ≤ 0.8 to 5 µm, with a majority of cells clustered around 2 µm, while P3 and P6 were characterized by cells of 0.8–2 µm and P4 by cells of 2–3.5 µm. Cells identified within P5 were larger, ranging from 3.5 to > 5 µm, therefore encompassing small-sized members of the nanoplankton (3–20 µm). Studies that provide size calibrations for sorted picoeukaryote populations are rare^37^, making direct comparisons unreliable.

The different small pigmented eukaryote populations had variable cell abundances relative to each other and varied with sampling location (Table 1). Surface communities were either dominated by population P3 (57–74% of small pigmented eukaryote abundance, hereafter counts) in the WPM (S003, S031 cast 03 (henceforth S031_03), and S031 cast 11 (henceforth S031_11)) as well as one station from EPM (S022) and one from OSW (S020), or by P2 (52–66% of counts) at the OPC (S024) and stations of the EPM (S025) and OSW (S027). All stations had lower abundances of P4 (5.7–32% of counts), and only four stations (S003, S020, S022, S031_11) also presented a small P5 population (3.1–6.3% of counts). The small pigmented eukaryote communities collected from chlorophyll maxima were all dominated by P2 (69–94% of counts) and accompanied by much less abundant P3 (5.6–23% of counts), except for station S022 whose chlorophyll maximum small pigmented eukaryote community was dominated by P3 (93% of counts). All chlorophyll maximum communities were characterized by a low contribution of P4 (0.6–6.2% of counts). The small pigmented eukaryote community collected from 40 m at S017 was characterized by the presence of population P6 (16% of counts), absent from the other stations. P1 was only present at the chlorophyll maximum of the OPC station (11% of counts). However, the amount of DNA extracted from P1 was too small to allow for sequencing of the 18S rDNA and it is therefore not part of the subsequent analyses.

**Table 1:** Cell counts per population, as the proportion of the summed total cell density for all 6 gated populations. CM, chlorophyll maximum.

Sample			Populations (as % of total)						Total (cells/mL)
Station		Depth	P1	P2	P3	P4	P5	P6	
**3**	3 m	Surface	0	18.63	57.23	18.24	5.90	0	677
**17**	40 m	Deep	0	53.41	13.17	16.22	1.16	16.04	406
**20**	3 m	Surface	0	4.08	73.95	15.68	6.28	0	2415
**22**	3 m	Surface	0	7.22	68.22	20.77	3.78	0	2337
	25 m	Subsurface	0	6.62	66.29	21.95	5.13	0	2374
	50 m	CM	0	1.15	92.62	6.23	0	0	4092
**24**	3 m	Surface	0	64.32	26.19	9.49	0	0	1707
	15 m	CM	10.73	77.40	17.22	5.38	0	0	1060
	30 m	Below- CM	0	1.21	98.23	0.56	0	0	2803
	60 m	Deep	0	0.79	95.92	3.29	0	0	1021
**25**	3 m	Surface	0	52.40	28.85	18.75	0	0	2246
	35 m	CM	0	82.50	15.97	1.53	0	0	529
**27**	4 m	Surface	0	66.12	28.15	5.73	0	0	1205
	90 m	CM	0	93.89	5.56	0.55	0	0	3524
**031_03**	3 m	Surface	0	10.17	57.36	32.48	0	0	1367
	27 m	CM	0	82.17	16.79	1.04	0	0	2338
**031_11**	3 m	Surface	0	5.99	72.19	18.72	3.10	0	2451
	27 m	CM	0	69.95	22.83	4.30	2.93	0	1456

At the surface, small pigmented eukaryotes contributed on average 1.3 ± 0.4% of the total small phytoplankton abundance (Supplementary Table S1), indicating that picocyanobacteria dominated all stations. *Synechococcus* dominated cell abundances at most stations (57–97%), except in the OSW (S020, S027) where *Prochlorococcus* dominated (92–97%). These results reflect the established paradigm that the eukaryotic component of small phytoplankton communities is less abundant than the prokaryotic component^10,16,37^. Nonetheless, in terms of biomass, small pigmented eukaryote dominated the small phytoplankton in all surface samples (11–44 × 10^3^ µg C/m^3^; 47–71%), representing a biomass greater than or equal to the picocyanobacteria (Supplementary Table S2). The horizontal shift in surface nutrient concentrations among habitats was too modest to affect the relative contribution of small pigmented eukaryote to total small phytoplankton abundances, contrary to reports for much larger spatial scales involving greater differences in nutrient concentrations, ranging from coastal systems to the open ocean^10,16^. Small photosynthetic eukaryote abundance and biomass were not significantly correlated to nutrient concentrations, salinity or temperature (Spearman rho < 0.4, *p *values > 0.05).

At the chlorophyll maxima and in deeper waters, despite a consistent predominance of *Prochlorococcus*, small pigmented eukaryotes generally contributed more to the total small phytoplankton abundance than at the surface, similar to previous reports from the Indian Ocean^7^ and the south Pacific Ocean^8,9^. These samples showed decreased absolute abundances of ≤ 5 µm phytoplankton (Supplementary Table S1), with the plume core stations (S017, S024) exhibiting the lowest overall absolute abundances (8.2–32 × 10^3^ cells/mL). This decrease in absolute numbers of the picocyanobacteria, concomitant with increased small pigmented eukaryote relative abundances (6–18%), indicated that eukaryotes fared better than the picocyanobacteria in the low light conditions of waters shaded by the plume. Dominance of the small phytoplankton biomass by small eukaryotes (5.9–50 µg C/m^3^; 53–95% of the total biomass), representing more than twice the picocyanobacterial biomass at the chlorophyll maxima and deeper water, is reminiscent of reports that small pigmented eukaryotes can contribute significantly to primary production in coastal regions^12^. Although this contrasts with findings from open oceans where *Prochlorococcus* dominates small phytoplankton biomass^11,37^, small pigmented eukaryotes were found to be similarly biomass-dominant and contributing up to a third of total primary production in surface seawater of the western subtropical North Atlantic under phosphorus depletion^13^.

### Taxonomic composition of small pigmented eukaryote populations

High-throughput sequencing of the small ribosomal subunit gene provided insights into the taxonomic composition of resident (live, inactive and recently dead) small pigmented eukaryote populations. A total of 234 operational taxonomic units (OTUs) were obtained, covering the full diversity of the populations in each sample (Supplementary Fig. S3) and after removal of metazoan OTUs and OTUs < 1% total reads, the final OTU count was 201 with variable distributions per population per sample (Table 2). Compared to protist communities collected and size-fractionated by filtration, reportedly composed of > 1,000 OTUs^38–42^, the low OTU richness is a reminder that our cell sorting protocol allowed the focused targeting of small pigmented eukaryote populations. The low OTU counts (3–42) for each population (Table 2) further reflect the accuracy of the sorting method and the near taxonomic purity of some of the sorted populations.

**Table 2:** Operational taxonomic units (OTUs) counts per population. CM, chlorophyll maximum.

Sample			Population				
Station		Depth	P2	P3	P4	P5	P6
**3**	3 m	Surface	24	10	12	–	–
**17**	40 m	Deep	9	8	7	–	5
**20**	3 m	Surface	18	19	18	19	–
**22**	3 m	Surface	14	17	7	12	–
	25 m	Subsurface	10	16	9	10	–
	50 m	CM	20	–	–	–	–
**24**	3 m	Surface	8	7	8	–	–
	15 m	CM	17	16	19	–	–
	30 m	Below—CM	9	10	–	–	–
	60 m	Deep	14	14	–	–	–
**25**	3 m	Surface	7	20	10	–	–
	35 m	CM	18	43	19	–	–
**27**	4 m	Surface	14	15	14	–	–
	90 m	CM	42	35	20	–	–
**031_03**	3 m	Surface	18	21	9	–	–
	27 m	CM	31	31	18	–	–
**031_11**	3 m	Surface	5	40	10	3	–
	27 m	CM	32	24	13	6	–
**33**	2 m	Surface	13	12	9	–	–
	17 m	CM	11	16	–	–	–

**Figure 3:**
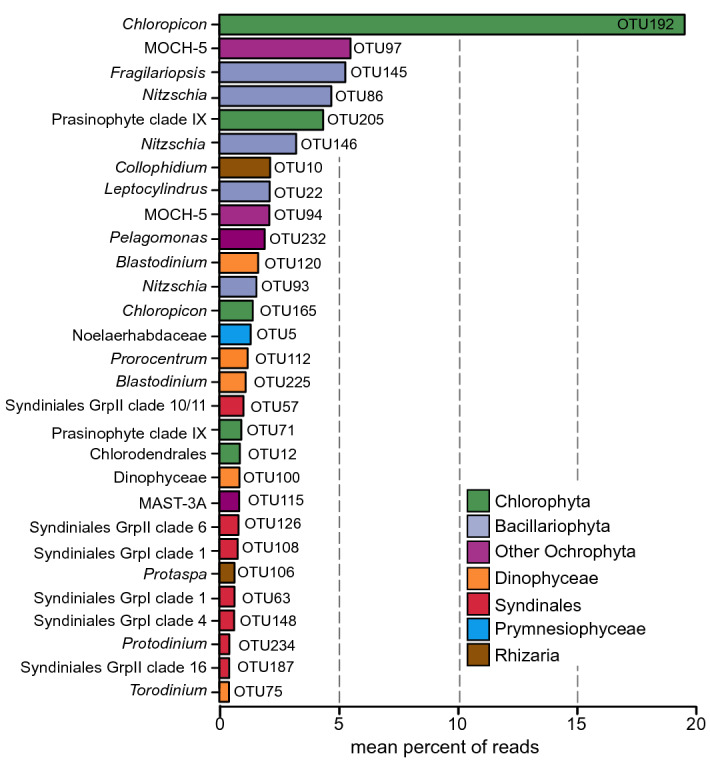
Barplot representing the average proportion of each of the 29 major OTUs distributed across all samples and all populations. Note that the major OTUs constitute at least 20% of the total reads per sample for at least one sample.

Major OTUs, constituting at least 20% of the total reads per population for at least one sample, represented 29 out of the 201 OTUs (Fig. 3; Supplementary Table S1). The most frequent OTU was a Chloropicophyceae (Chlorophyta), averaging 19.55% of total reads/sample. The second most frequent Chlorophyta OTU belonged to prasinophyte clade IX with an average of 4.36% of the total reads/sample. The Ochrophyta were represented by two Marine Ochrophyta clade 5 (MOCH-5) OTUs and five Bacillariophyceae OTUs ranging on average from 5.5 to 2.1%, and 5.3 to 1.6% of total reads per sample, respectively. Only one major OTU was associated with the prymnesiophytes, classified within the order Isochrysidales, representing 1.3% of the total reads/sample (Fig. 3). The rest of the major OTUs had lower average abundances throughout the samples (< 2%) reflecting a more localized dominance (Fig. 3). Taxa represented by these OTUs were most frequently assigned to the Dinophyceae and the Pelagophyceae.

**Figure 4:**
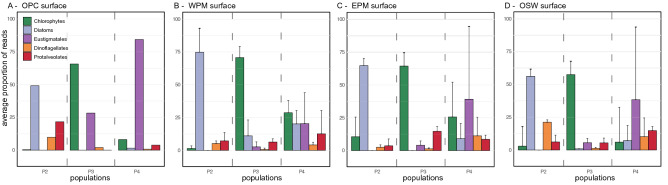
Barplot of the five most abundant taxa, per population for each habitat type in surface samples. OPC, old plume core; WPM, west plume margin, EPM, east plume margin; OSW, ocean seawater. Error bars indicate one-sided standard deviation among samples of the same habitat type. Absence of error bars when there was only one sample per habitat type.

The three most frequent algal groups dominated different populations (Fig. 4). The most abundant populations, P2 and P3, were dominated by Bacillariophyta (diatoms) and Chlorophyta, respectively. Diatoms usually characterize microphytoplankton communities, especially in nutrient rich regions^43^, but members of the Bacillariophyta, including *Chaetoceros, Thalassiosira, Pseudonitzschia, Skeletonema* and *Minidiscus,* present a size range of the 2–5 µm^24,26,44–48^. The most frequent diatom OTUs throughout the five ARP habitats were closely related to *Fragilariopsis* (OTU145) and *Nitzschia* (OTU86 and OTU146), as confirmed by evolutionary placement analysis (Supplementary Fig. S5). Diatoms ≤ 5 µm have been reported from various oceanic regions^24,49,50^, and can represent a large part of the small pigmented eukaryote communities, as evidenced in the global *Tara Oceans* dataset^24^.

*Nitzschia* OTUs constituted half the OTU counts in the largest P2 populations, which reached 1.0–1.2 × 10^3^ cell/mL at the surface in the OPC and one EPM station, and 1.0–3.3 × 10^3^ cell/mL at the chlorophyll maximum in two WPM stations and one OSW station, showing that Bacillariophyta ≤ 3.5 µm were abundant in these samples. In general, Bacillariophyta ≤ 5 µm are chronically under sampled and misidentified and may form blooms more frequently than expected, particularly in turbulent nutrient-rich areas caused by mixing or frontal stirring^24^. Small diatoms have been unnoticed in the ARP region, where past studies have focused on ≥ 10 µm diatoms which may form symbioses with diazotrophic cyanobacteria^1,3,5,6^. However, our study suggests that there is a niche for smaller diatoms in the old plume (S024), possibly extending to the plume margins (S025). These siliceous organisms can easily aggregate, resulting in higher sinking rates than generally assumed for this size class^24,51^. Hence, the rates of carbon sequestration driven by the ARP, which have heretofore been based on larger diatoms and diatom-diazotroph associations^3,4^, have likely been underestimated.

**Figure 5:**
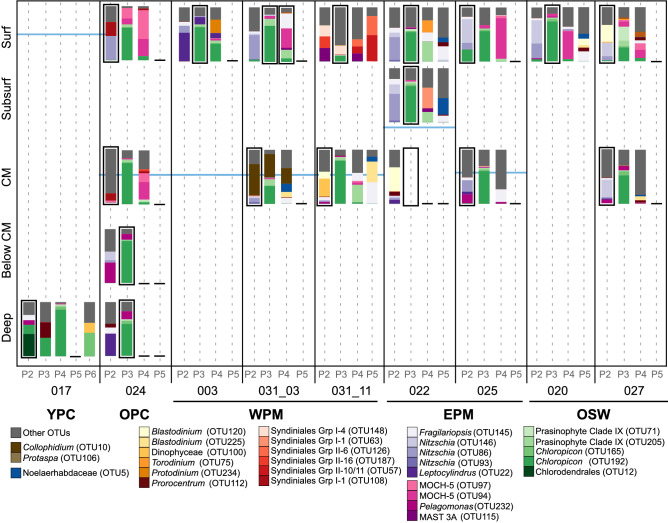
Relative contribution of the major OTUs to each population. OTU compositions are represented as barplots. The most abundant population in each sample is indicated by a black border around the corresponding barplot. Note that the sample for cell counts at S033 was lost, so no data are shown for this sample. Empty barplots indicate missing 18S rDNA data due to low DNA concentrations or low quality reads. The blue lines indicate the position of the halocline. “Other OTUs” indicate OTUs that only contributed less than 20% of a population in any given sample. Grp., group; OTU, operational taxonomic unit; MOCH, Marine Ochrophyta; MAST, Marine Stramenopile.

Small Chlorophyta tend to be abundant in nutrient rich coastal waters^19,52,53^, but are also found in oligotrophic regions, such as open oceans^8,54,55^. Notably, they dominate the picophytoplankton in the Arctic Ocean^27,56^. The Chlorophyta reads found in population P3 belonged to three important classes/clades. The Mamiellophyceae, a Chlorophyta group that dominates in coastal waters^15,20,25^, were rare in the ARP and western tropical North Atlantic Ocean with infrequent low abundance OTUs related to *Bathycoccus* and *Micromonas* (Supplementary Table S4). Prasinophyte clade IX was represented by two major OTUs (Fig. 3) with widespread distribution among the ARP habitats, one of which was found in highest abundance at S022 (EPM) in P4 (Fig. 5). Although there is no known cultured representative, this clade is regularly found to contribute significantly to the community composition in open-ocean water^15,57^. Chloropicophyceae, previously referred to as prasinophyte clade VII^58^, was ubiquitous with the most frequently detected major OTU, *Chloropicon* OTU192. This OTU branched close to Prasinophyceae sp*.* RCC917 (Supplementary Fig. S6), a representative of the species *Chloropicon roscoffensis*^58^, isolated from the Southeast Pacific Ocean^59^. The dominance of Chloropicophyceae is consistent with reports of small pigmented eukaryote communities in moderately oligotrophic conditions^15,57,60^ as well as in coastal waters^8,19^.

The OTU composition of P4 was more variable between stations than other populations, although it was repeatedly enriched with MOCH-5 (Figs. 3, 5). This group is a recently identified Stramenopile ribotype, presumed to be a novel algal lineage^61^. Although widely distributed in the Pacific Ocean^62,63^, the Atlantic Ocean^63^ and Mediterranean Sea^64^, MOCH-5 is generally not reported to be an abundant part of the phototrophic protist communities. Haptophyta OTUs were in low abundance, detected in P2 and P4 populations at most stations (0.2–8.1%; Supplementary Table S4). Haptophyta, and Prymnesiophyceae in particular, are reported to contribute significantly to diversity and biomass among small pigmented eukaryote communities of open oceans, including oligotrophic gyres^16,21,65,66^, therein contributing significantly to primary production^37^. Unexpectedly, Prymnesiophyceae were represented only by one major OTU, attributed to the Isochrysidales (OTU5), detected exclusively among the P5 population, at station S022. This result contrasts with the significant haptophyta populations previously detected in the ARP margins during the summer^6^. However, that study was based on collection of phytoplankton with an 8 µm pore-size filter and as the largest size class of our study was 3.5–5 µm (P5), the comparatively underwhelming presence of haptophyta OTUs in our dataset is likely due to the exclusion of > 8 µm cells from our sorted populations. Furthermore, the consistently low abundance or absence of P5 throughout our samples suggests that this Isochrysidales OTU5 (Noelaerhabdaceae) did not dominate the small pigmented eukaryote communities of the ARP in the spring.

### Distinguishing the small pigmented eukaryote community composition between habitat types

**Figure 6:**
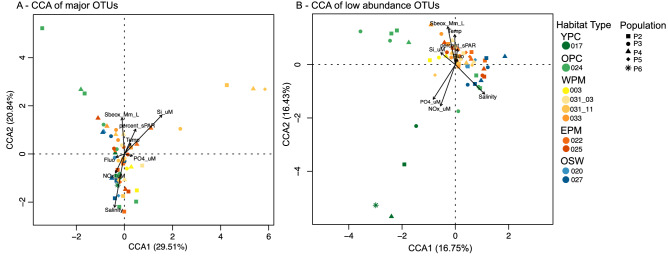
Canonical correspondence analysis with A major OTUs, and B low abundance OTUs.

A UniFrac unweighted paired group method with arithmetic mean analysis revealed stronger clustering among populations than among stations or depths, suggesting a consistency in the phylogenetic composition of the sorted populations (Supplementary Fig. S4). The only exceptions were S031_03 chlorophyll maximum and S031_11 surface samples for which the three populations clustered distinctly from the rest. A canonical correspondence analysis based on assemblages of major OTUs separated populations P2 and P4 of the OPC surface and subsurface and all four populations of S031_11 surface from the rest of the samples (Fig. 6a). The low abundance OTU composition of the surface and subsurface OPC populations and the deep YPC sample were distinct from the rest of the samples, the latter being strongly driven by salinity (Fig. 6b). The environmental variables used in the canonical correspondence analysis explain a sizable portion of the variability (33–50%), although it seems that an important driver of community composition was unaccounted for.

The YPC populations had low OTU richness (Table 2), and most of their major OTUs were shared with other stations, namely the Chloropicophyceae OTU192 and OTU165, detected in all 4 populations (16–86% of total reads/population). Notably, this sample was only distinguished from the rest by its low abundance OTU composition (Fig. 6b). Of the 15 low-abundance OTUs among the 4 populations detected, a few were shared with other samples, but only one or two at a time (Supplementary Table S3). The OPC surface was also characterized by a low OTU richness (Table 2), each population dominated by one or two major OTUs (Fig. 5). P2 was dominated by Bacillariophyta *Nitzschia* (OTU86), also found at other stations in lower abundance, and by a Syndiniales GrpI OTU108 unique to this station. P3 was composed of the ubiquitous Chloropicophyceae OTU192, classified as *Chloropicon,* and two MOCH-5 OTUs, which were also found in P4. The chlorophyll maximum sample was composed of a very similar small pigmented eukaryote community, albeit with a larger proportion of low abundance OTUs in P2. Interestingly, P3 at both surface and chlorophyll maximum was distinguished from other samples by the low abundance OTUs that accompanied the dominant Chloropicophyceae OTUs (Fig. 6b). The small pigmented eukaryote community of the sample below the chlorophyll maximum was characterized by an abundant P3 dominated by *Chloropicon* OTU192, accompanied by the Pelagophyceae *Pelagomonas* OTU232, which was also detected at S025 (EPM) and S027 (OSW). This sample collected below the halocline was distinct from the upper water column and more similar to the margin and oceanic samples. Such a pattern is consistent with the plume overriding the surrounding margin or oceanic waters and submerging the endemic communities that were there previously at the surface.

In contrast to our first hypothesis regarding small pigmented eukaryote variability across the horizontal gradients of the ARP, the composition of small pigmented eukaryote communities was stable among the different habitat types. This is attributable to a combination of variability in OTU composition among samples from the same habitats and similarity of the small pigmented eukaryote assemblages between stations of different habitats. Indeed, the populations exhibited no significant differences between average UniFrac distances among habitats, stations of the same habitats and depths of the same stations (ANOVA, *p* > 0.164 for P2, *p* > 0.251 for P3 and *p* > 0.735 for P4). The lack of statistical differences, particularly among the plume margins and oceanic waters, are indicative of the dynamic nature of large river plumes, such as reported for the Columbia River^67^. The meandering of the ARP creates a very dynamic system with a variable influence on local oligotrophic ocean waters^68,69^. It is possible that each station is too unique to establish a consensus small pigmented eukaryote community structure per habitat type, while abundant populations are shared between stations of different habitats limiting the detectable distinctions between the assemblages. For instance, the dominant *Nitzschia* OTU86 was shared between the OPC, one of the WPM stations and one of the EPM stations. Similarly, *Chloropicon* OTU192 dominated P3 at all stations, except in the surface waters of one WPM station (S031_11) and one OSW station (S027). Furthermore, our use of DNA as template for the taxonomic survey might have masked changes in the active communities among different habitats that would have been more apparent with RNA templates.

The progressive mixing of oceanic waters into the plume is likely to exchange small pigmented eukaryote communities between the adjacent environments. This hydrodynamic phenomenon would allow the unrestrained dispersal of small pigmented eukaryotes between habitats, resulting in the observed similarities between the plume and surrounding ocean surface waters. In the dynamic environment of the ARP margins, the similarity between communities of different habitats is a function of time since the onset of the mixing event that exposed oceanic and plume small pigmented eukaryote communities to adjacent environments. Time-since-mixing might be the environmental parameter unaccounted for in our dataset that would explain the intra-habitat variability in major OTU composition, incidentally, obscuring the differences between habitats.

Contrary to picocyanobacteria, which mostly use recycled, reduced forms of nitrogen (ammonium and urea), small pigmented eukaryotes rely more on nitrate^70,71^, making them more sensitive to the low nitrate concentrations in and around the ARP. While the uniformity of small pigmented eukaryote biomass between the oligotrophic ocean waters and the plume margins is likely the product of low nutrient concentrations in both environments, the variability of the OTU composition might be explained by a variable nitrate metabolism among small pigmented eukaryote taxa^70^. Alternatively, mixotrophy, the combination of photosynthesis and bacterivory common among small pigmented eukaryotes^13,72–74^, might confer a generalist advantage relative to picocyanobacteria by allowing maintenance of activity and abundance in rapidly varying habitats.

Corroborating our second hypothesis that the small pigmented eukaryote diversity should vary with depth within the euphotic layer, the small pigmented eukaryote diversity and abundance varied vertically, with higher cell counts at the chlorophyll maximum. The taxonomic composition of chlorophyll maximum communities differed from those at the surface with populations characterized by high abundances of OTUs associated with Bacillariophecae, Pelagophyceae, radiolarians or Dinophyceae. The presence of Dinophyceae or Pelagophyceae OTUs at the chlorophyll maxima of plume stations (OPC, WPM and EPM), which were absent from surface waters, reflects the strong stratification at plume-influenced stations, reducing mixing between the surface and the bottom of the euphotic zone, the latter of which can be strongly influenced by oceanic waters. In particular at these stations (S024, S031 and S022), the chlorophyll maximum samples were collected below the halocline depth. Hence, these Dinophyceae and Pelagophyceae OTUs, uniquely shared with one of the oceanic stations, suggest that water masses under the plume-influenced surface might correspond to the oceanic water masses at the OSW stations.

Station S031, a time-series station, showed a variation in major OTU assemblages between the cast conducted at 3 pm on May 26th (S031_03) and another cast carried out at 11am on May 27th (S031_11). Within this 19-h interval, in which environmental conditions remained consistent with the habitat type (Fig. 1), the OTU composition underwent a shift (Figs. 5, 6a). The relative cell abundances of each population remained similar, except for a P5 population appearing in samples from the second time point (Fig. 5). At the surface, the shift was characterized by the replacement of all OTUs from S031_03 with major OTUs assigned to Syndiniales in S031_11. The only common OTU, MAST-3A (OTU115), had low abundances (0.4–3.7%) in S031_03 and reached 14–24% in the S031_11 populations (Supplementary Table S3). Interestingly, the major Syndiniales OTUs in S031_11 were unique to this station, and different from the Syndiniales OTUs detected in the OPC and EPM stations (Fig. 5). This unexpected abundance in unpigmented Syndiniales OTUs in S031_11 might be due to the presence of dinospores in transitory free-living form, attached to or inside alveolate hosts or predators^75,76^. The large proportion of low abundance OTUs, which represented 70% of total reads in P3, were related to Syndiniales, ciliates and dinoflagellates (Supplementary Material SM2).

Changes were also observed at the chlorophyll maximum where the unique radiolarian *Collophidium* OTU that dominated S031_03 disappeared in S031_11. This abundance of sequences related to the Radiolaria, large heterotrophic protozoa (≥ 100 µm), was unexpected among our targeted populations sorted by size and chlorophyll content. However, radiolarian sequences have been found among small size fractions before^77,78^, particularly at depth^79,80^ where they are suspected to descend and release small flagellate gametes called swarmers^81^. Hence, if attached to exopolymer-producing pigmented cells such as in the late stages of a phytoplankton bloom^82^, these swarmers could have been indiscriminately sorted into the three populations. In addition, three dinoflagellate OTUs appeared in P2 and P4 in cast S031_11, of which one was only found in the deep YPC, and one was shared with the chlorophyll maximum of S022 (EPM) and the surface of S027 (OSW).

The radical shift in small pigmented eukaryote community composition between the two casts from station S031 reflects the dynamic nature of the ARP ecosystem and the multiple scales of heterogeneity within this system that is unlikely to be uncovered without the multiple approaches used in this study. It is unlikely that this interval of 19 h was sufficient for the resident small pigmented eukaryote community to change so radically as to completely replace the original taxa, as taxonomic turnover on daily time scales is usually very limited^83,84^. The salinity profiles indicated a stronger stratification at the time of cast 11, with a deeper mixing depth (22 m) compared to cast 03 (16 m), reflected in the chlorophyll profiles showing more homogenous concentrations in the top 22 m of cast 11 (Supplementary Fig. S1). In addition, the chlorophyll maximum peak sampled at 27 m was much smaller in cast 11 compared to cast 03, with a stronger secondary peak at 39 m. Satellite observations show the river plume defined as high surface chlorophyll, spreading north and eastward between the 25th and 29th of May (Supplementary Fig. S9—higher chlorophyll concentrations north of 17°N), suggesting a plume that was advecting past the ship during this time. This likely caused the deepening of the mixing depth, forcing the surface small pigmented eukaryote community northwards and the chlorophyll maximum community deeper below the mixing depth, effectively displacing the communities identified during cast 03.

As a first study of the small pigmented eukaryotes and their response to the environmental habitats of the ARP, this work provides new insights into the detailed 18S rDNA-based taxonomy of an underexplored fraction of the phytoplankton. Our results illustrate that FACS is a reliable tool to enrich targeted taxonomic groups, such as Bacillariophyta, Chlorophyta and MOCH-5. The small pigmented eukaryote taxonomic composition was influenced by the ARP only at the plume core (OPC) where surface assemblages showed a strong dissimilarity with other stations, which were otherwise similar despite belonging to different habitat types. This result stands in apparent contrast to the drastic succession in community composition of the microphytoplankton driven by the nutrient gradients in the ARP^1,3,4,6^. The surprisingly limited influence of the ARP on surface small pigmented eukaryote communities warrants further inquiry. Sampling at different times of the year and using 18S rRNA as template for sequencing might reveal small pigmented eukaryotes to be more reactive to the habitat types earlier in the season, at the beginning of the massive discharge period from the Amazon River, or at the end of the summer when the ARP is entrained toward the east by the north equatorial countercurrent.

## Materials and methods

### Sampling

Seawater samples were collected onboard the *R/V Endeavor* during cruise EN614 in the western tropical North Atlantic in May 2018, following surface salinity as indicator for proximity to the plume (Fig. 1c). We received clearance to sample in the EEZs of Guyana, Suriname, and French Guiana (US Department of State Clearance # 2018–1,241,589). The thermohaline water masses in and around the ARP were identified as North Atlantic central waters (Temperature < 20 °C, Salinity > 35), tropical surface waters (T > 20 °C, Sal > 34), coastal waters (T > 25 °C, Sal = 30–24), and plume waters (T > 25 °C, Sal ≤ 30). Seawater, collected using Niskin bottles attached to a CTD-rosette, was sampled in acid-washed carboys kept in a light-proof bag, then prepared for preservation within 30 min.

For quantitative cell counts using flow cytometry, 4.5 mL of seawater was mixed with 140 µL of PFA 32%, incubated in the dark for 15 min, flash-frozen in liquid nitrogen and stored at -80 °C until processing. To concentrate microbial populations for FACS, 1 L of seawater was gently filtered onto a 0.2 µm PC membrane (Millipore) using a peristaltic pump. The filter was transferred into a 5 mL cryovial containing 4 mL of seawater sample from the same station and depth and 0.5 mL of TE-glycerol^85^. The cryovial was vortexed for 1 min to resuspend a maximum of cells and flash-frozen in liquid nitrogen for storage at -80 °C until processing. Seawater for dissolved nutrient concentrations was collected from the same depths and analyzed on-board using a Lachat QuikChem 8000 flow-injection analysis system.

### Habitat types

We combined data from fifteen stations collectively sampled during the EN614 cruise, among which ten were sampled for our small pigmented eukaryote study (Fig. 1). Habitat types are defined by the correlation of five environmental variables; sea surface salinity, sea surface temperature, a nitrogen availability index, the mixed layer depth and the chlorophyll maximum depth as defined by Weber et al.^35^. The latter three are integrated parameters, derived from vertical profiles of dissolved nitrogen concentrations, buoyancy frequency, and chlorophyll fluorescence, respectively (Supplementary Fig. S1, S8). The buoyancy frequency, or Brunt–Väisälä frequency, was calculated with the swN2 function of the oce package in R. Chlorophyll concentration was estimated via fluorescence measurements, using a CTD fluorometer calibrated in the factory on 2 March 2018, just before the cruise. The principal component analysis and Ward’s hierarchical cluster analysis were carried out with the vegan package^86^ in R^87^.

### Cell counts and cell sorting

Small pigmented eukaryote cell counts and cell sorting were conducted as previously described^9,13^ using a BD Influx flow cytometer, equipped with 70 µm nozzle and a blue laser (488 nm, 200 mW solid state). The sheath fluid consisted of a sodium chloride solution (6 g/L) filtered in-line through a 0.22-μm Sterivex filter unit. Reference beads (Fluoresbrite, yellow-green, 1-μm) were regularly added to a MilliQ control, between samples, to maintain proper alignment and focus of the instrument. Event collection was triggered based on the signal detected by the forward scatter (FSC) with small particle option. Careful gating in a trigger pulse width vs. FSC plot ensured that most aggregates and potentially attached cells were discarded. Small pigmented eukaryotes were discriminated based on their chlorophyll (red) fluorescence and FSC (size) signatures, excluding the heterotrophic components of the protist communities (Fig. 2a). Cyanobacterial cells were excluded based on phycoerythrin (orange) fluorescence (Fig. 2b). See Supplementary Material SM1 for a description of sorted populations of small pigmented eukaryotes. Polystyrene non-fluorescent beads of 0.79, 2, 3.39 and 5.11 µm (Flow Cytometry Size Standards Kit, Spherotech) were used to estimate the size range of each population.

Cytograms were analyzed with FCS Express 6 (De Novo software) to calculate cell abundances. Biomass of each population (in µg C/m^3^) was estimated based on the size estimates to obtain radius, assuming cell morphology to be similar to a sphere, and using a biovolume-carbon conversion factor of 237 fg C/µm^3^, as per Rii et al.^11^. A minimum of 8,000 cells per population^13^ were sorted into separated 2 mL microcentrifuge tubes containing 50 µL of PureLink Lysing Buffer (Invitrogen). The sorted populations were then vortexed briefly and stored at − 80 °C until DNA extractions.

### DNA extractions, PCRs and sequencing

Following an initial proteinase K lysing step at 50 °C for 3 h, DNA extractions were conducted with PureLink DNA mini kit (Invitrogen) according to manufacturer’s instructions. Polymerase chain reactions were conducted with partial-adapter primers 18S_0587_F (5′-ACA CTC TTT CCC TAC ACG ACG CTC TTC CGA TCT CCG CGG TAA TTC CAG CTC-3′) and 18S_0964_ R (5′-G ACT GGA GTT CAG ACG TGT GCT CTT CCG ATC TGA TCC CYY AAC TTT CGT TCT TGA-3′), targeting the V4 region of the 18S ribosomal RNA gene^40^. Amplicons were then purified with Ampure XP Purification magnetic beads and sent to GENEWIZ (South Plainfield, NJ) for sequencing. All populations were sequenced in three separate runs on Illumina HiSeq, according to the company’s automated EZ-Amplicon pipeline.

### Amplicon read analyses

Raw reads were deposited in the NCBI Short Read Archive (SRA) under the project accession number PRJNA701187. Analyses of 18S rDNA amplicon reads were carried out with Qiime2^88^. Denoising, dereplication, trimming and chimera check was done with q2-dada2. Closed-reference clustering was conducted against the SILVA 18S rRNA gene database release 128 using 97% similarity cut-off. Following de novo chimera identification and removal, abundance filtering was conducted to remove singleton OTUs and read counts were equalized to 2,000 reads per population. The subsequent taxonomic classification was based on a naïve Bayes trained PR2 v4.14 database^89^. OTUs with read count < 1% of the total (“rare” OTUs) and OTUs assigned to Metazoa were removed from the final OTU table, to reduce risk of background signal from cross-sorting of low-count representatives^13^. The OTUs were then separated into “major OTUs”, constituting at least 20% of the total reads per population for at least one sample, and the rest which were labeled “low abundance OTUs”.

Evolutionary placements were carried out for a better resolution of OTU classifications for Bacillariophyta, Chlorophyta, Dinoflagellata. Reference 18S rDNA sequences for each phylum were obtained from the NCBI Nucleotide database and aligned using MUSCLE (v3.8.31)^90^. Reference phylogenetic reconstructions and bootstrap analyses were carried out with RaxML-HPC (v7.2.8)^91^ using the GTRCAT model. The OTU reads were then evolutionarily placed into the reference tree using the RaxML-HPC EPA command and visualized in R using the ape package^92^.

### Maps and statistics

A map was created using the NASA SeaDAS 7.5.3 software (https://seadas.gsfc.nasa.gov), showing the monthly composite surface chlorophyll concentration for May 2018 was based on ESA satellite observations Reprocessed L4 downloaded from Copernicus Marine Service (https://resources.marine.copernicus.eu/?option=com_csw&view=details&product_id=OCEANCOLOUR_GLO_CHL_L4_REP_OBSERVATIONS_009_093). Land borders and exclusive economic zones (EEZ) were overlain on the resulting map using gmt v5.4.5^93^.

Normalization of the read counts per FACS sorted population was carried out to reliably compare the OTU composition among populations. OTU read counts were normalized to the different population sizes by multiplying the read counts by the number of cells counted for that population and divided by the total small, pigmented eukaryote counts for the sample. For beta-diversity analysis, Unifrac distances were estimated in Qiime2, based on a phylogenetic reconstruction with FastTree^94^, and represented by a unweighted paired group method with arithmetic mean analysis^95^ cluster dendrogram. The canonical component analysis was carried out in R-studio with the vegan^86^ and ape^92^ packages, following a Hellinger transformation^96^ of the OTU table.

## Supplementary Information

Supplementary Figures.

Supplementary Information.

Supplementary Table S1.

Supplementary Table S2.

Supplementary Table S3.

Supplementary Table S4.

## Data Availability

Raw reads and metadata were deposited in the NCBI Short Read Archive (SRA) under the project accession number PRJNA701187. A detailed workflow of command lines used is available in Supplementary Materials SM3 and the full OTU table can be made available by the authors upon request.
