# Supplementary material for: Small pigmented eukaryote assemblages of the western tropical North Atlantic around the Amazon River plume during spring discharge
Source: Sci Rep. 2021 Aug 10;11:16200. doi: 10.1038/s41598-021-95676-2 (PMC8355221; doi:10.1038/s41598-021-95676-2)
Supplement: Supplementary file 1 — Supplementary Figures. [file 41598_2021_95676_MOESM1_ESM.pdf]

Small pigmented eukaryote assemblages of the western tropical North Atlantic around the Amazon River plume during spring discharge.

Sophie Charvet, Eunsoo Kim, Ajit Subramaniam, Joseph Montoya and Solange Duhamel

Supplementary Figures

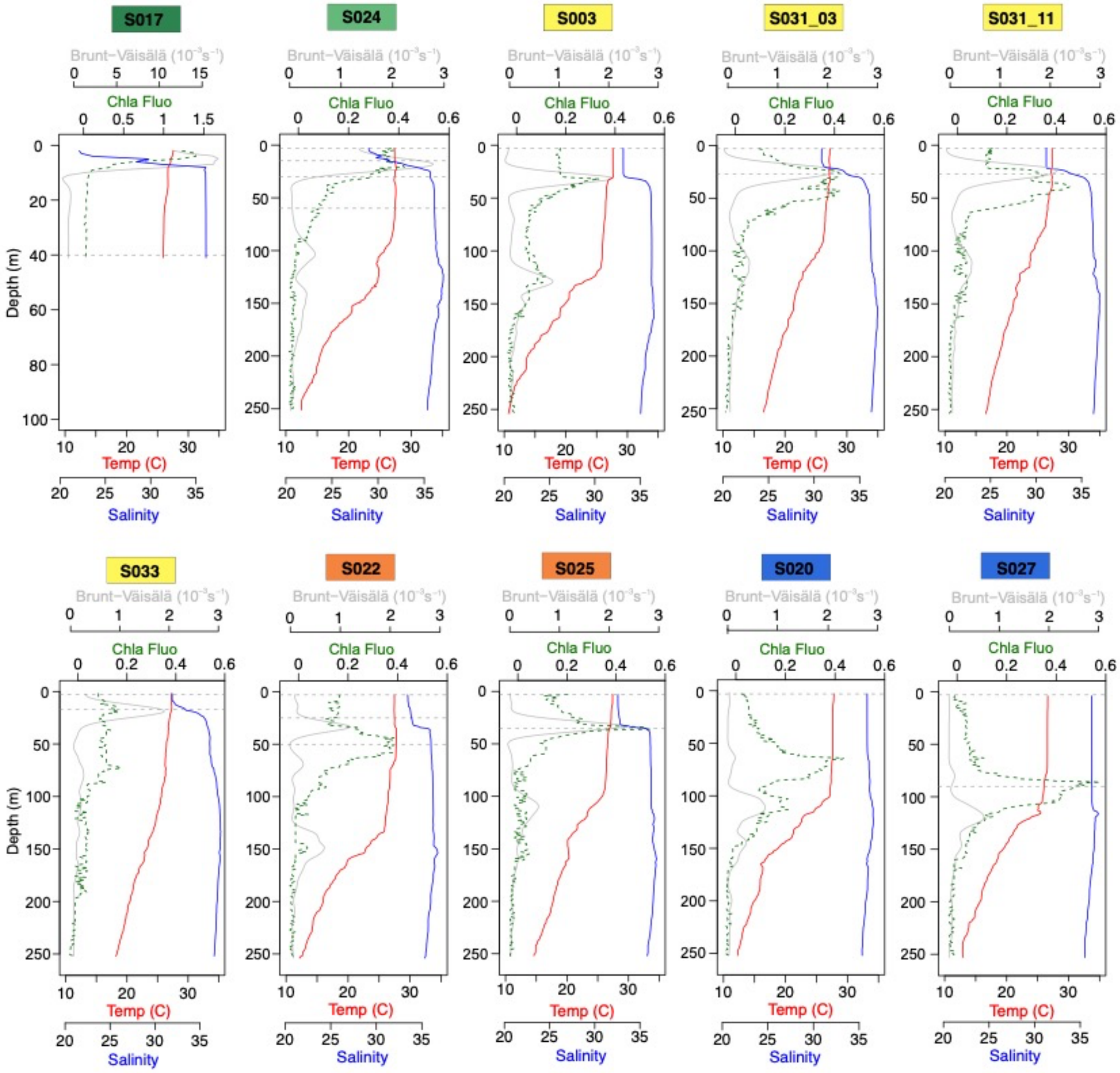

Supp. Fig. S1. Temperature, salinity, chlorophyll *a,* and buoyancy profiles at each station sampled. Note the different scales for chlorophyll *a* and the Brunt-Väisälä frequency at station S017. The grey dashed lines indicate the depths at which samples were collected for DNA analyses. Brunt-Väisälä corresponds to buoyancy frequency (radians per seconds, s^{-1}) Chla Fluo corresponds to chlorophyll *a* concentration (mg/m^3) estimated by fluorometry; Temp is temperature (°C).

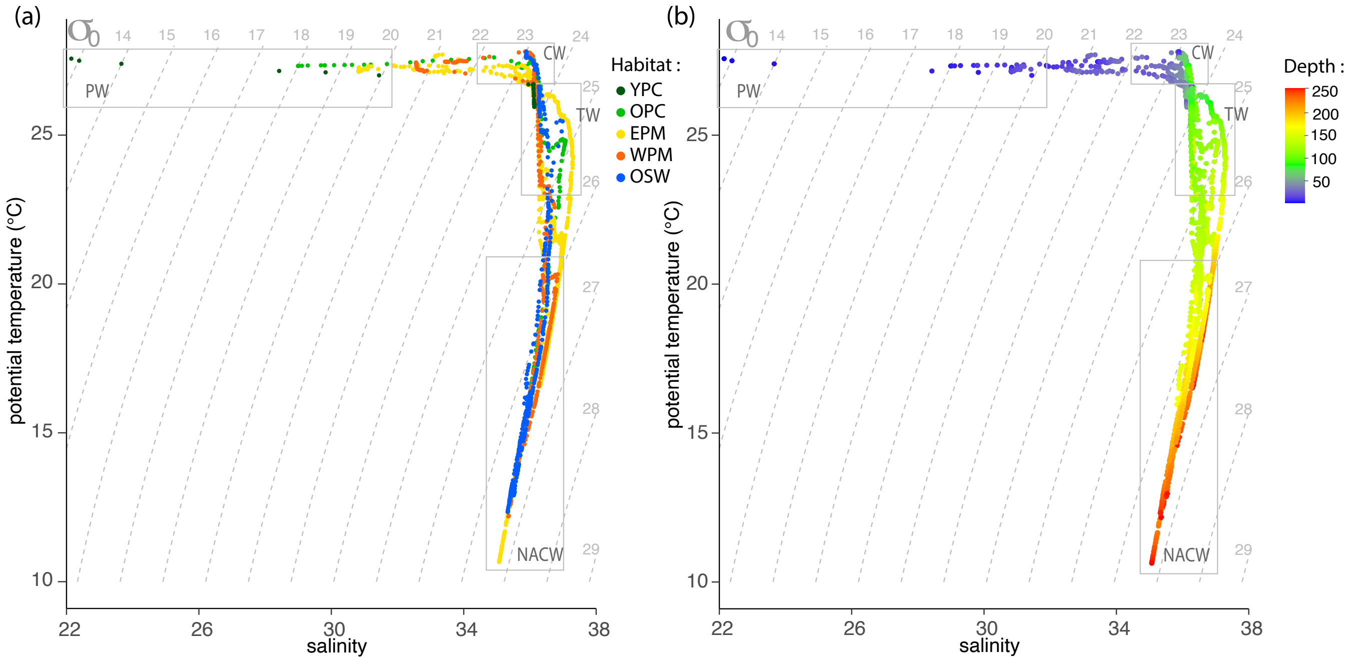

Supp. Fig. S2. Temperature-Salinity diagrams showing the distribution of stations across the water masses, color-coded according to habitat (a) or depth (b). Diagonal dashed lines represent the density isopycnals (σ_0; in mg cm^{-3}). Gray squares indicate the water masses: NACW, North Atlantic central waters; TW, tropical waters; CW, coastal waters; PW, plume waters. Habitat types: YPC, young plume core; OPC, old plume core, EPM, east plume margin; WPM, west plume margin; OSW, ocean seawater.

a)

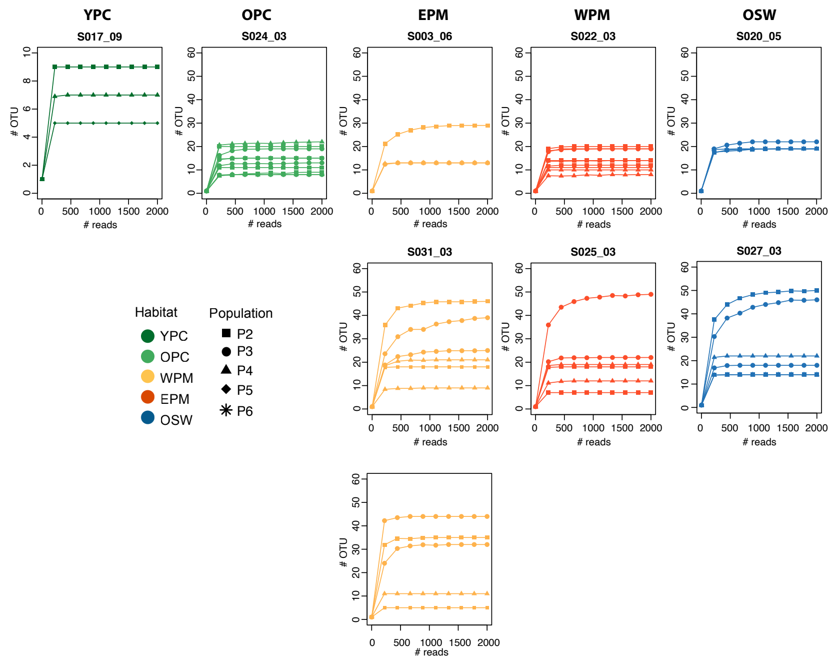

b)

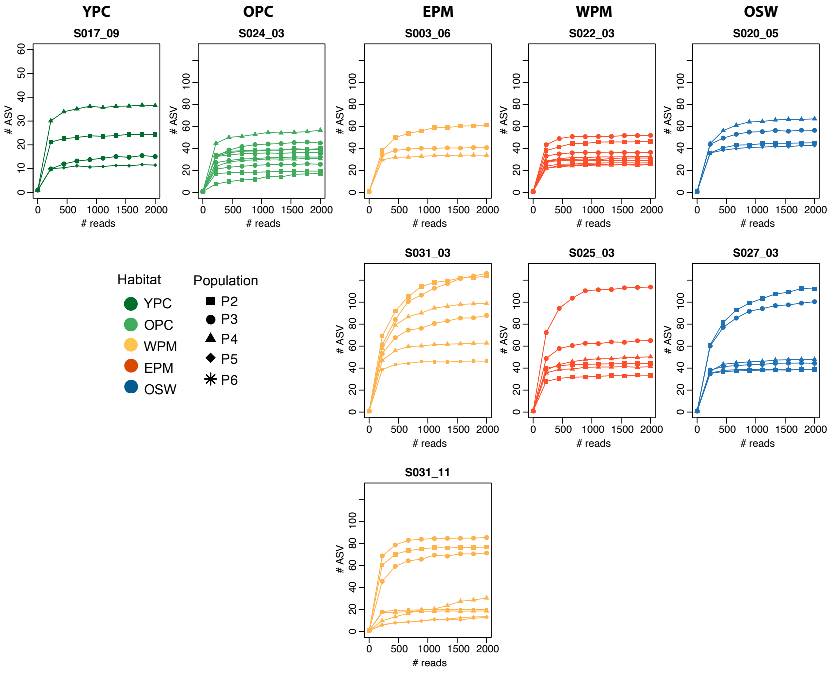

Supp. Figure S3: Rarefaction curves for (a) OTUs and (b) ASVs showing the diversity sampled by sequencing depth (represented as # reads). Note the different scales for S017. These rarefaction curves indicate that with 2000 reads per samples, the full diversity of the populations was adequately represented. OTU, operational taxonomic units; ASV, amplicon sequence variant.

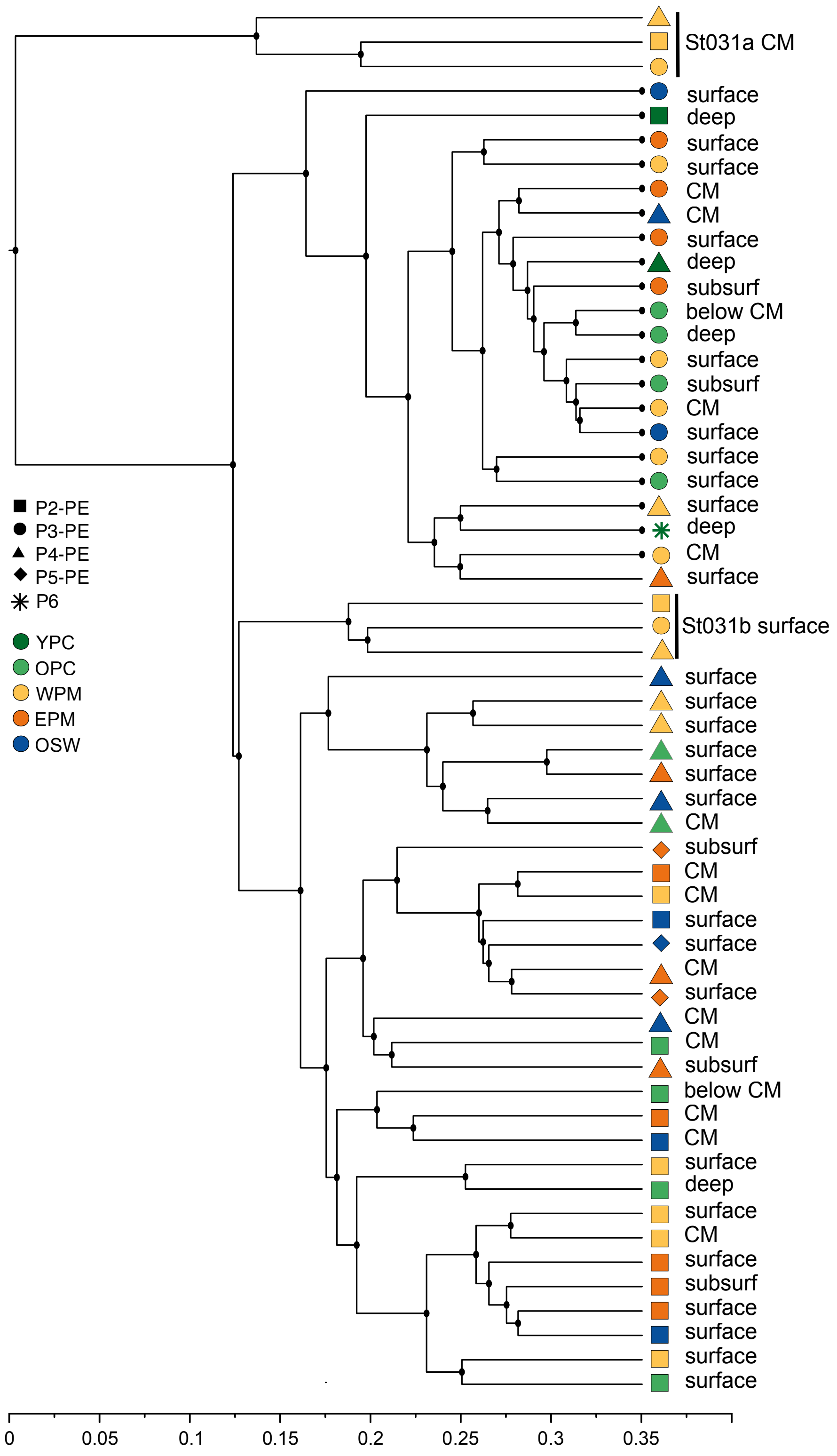

Supplementary Figure S4. UPGMA (unweighted pair group method with arithmetic mean) of weighted UniFrac distance between the populations of each sample. OPC, old plume core; WPM, west plume margin, EPM, east plume margin; OSW, ocean seawater.

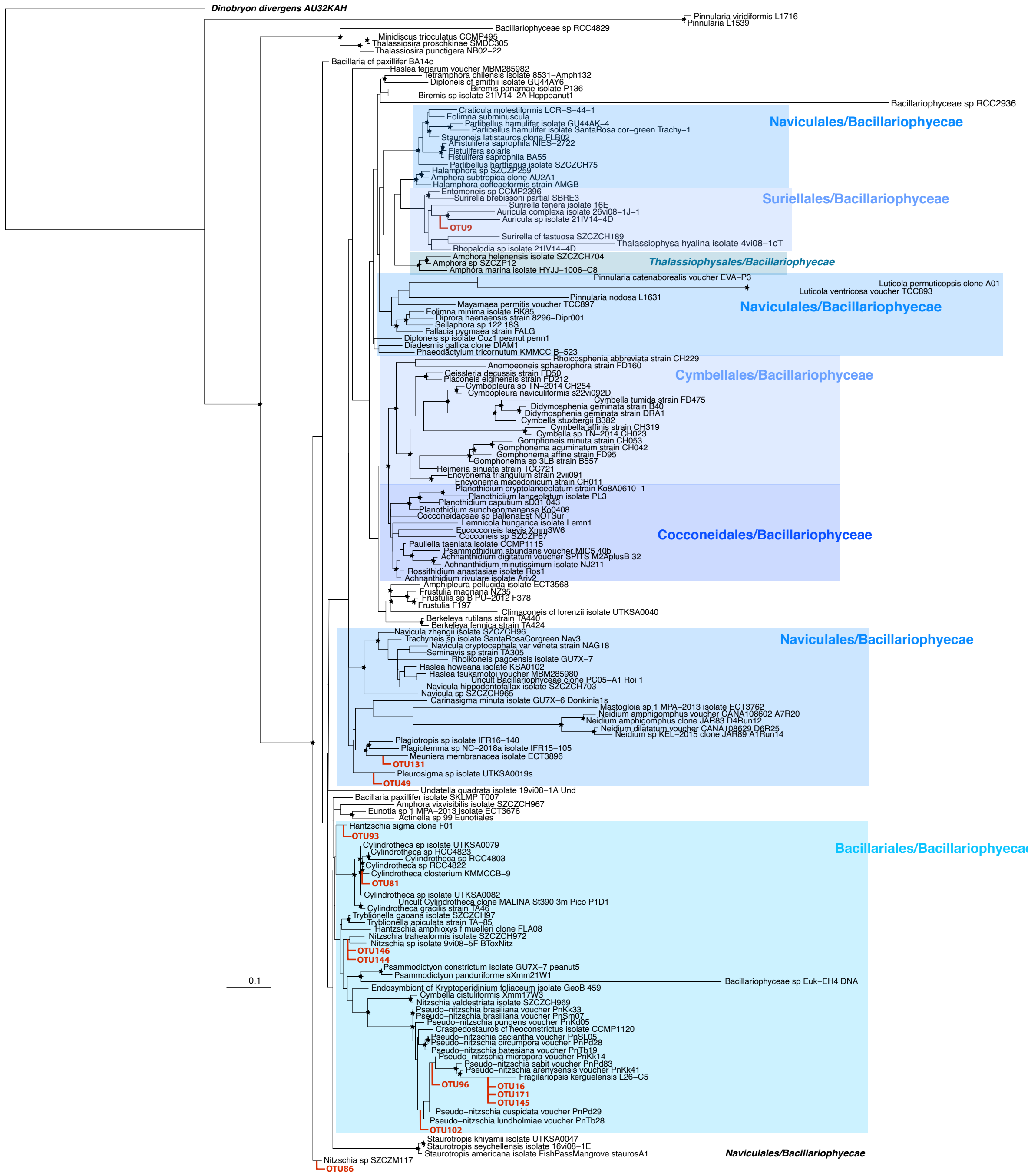

Supplementary Figure S5. Evolutionary placement analysis (EPA) of the Bacillariophyecae operational taxonomic units (OTUs). The reference tree is rooted with an outgroup from the chrysophytes, illustrated by *Dinobryon divergens* Nodes with bootstrap values above 50% are marked with stars. OTU placements are indicated red branches for recent branching placements. Note that the red branch lengths are not representative of phylogenetic distance.

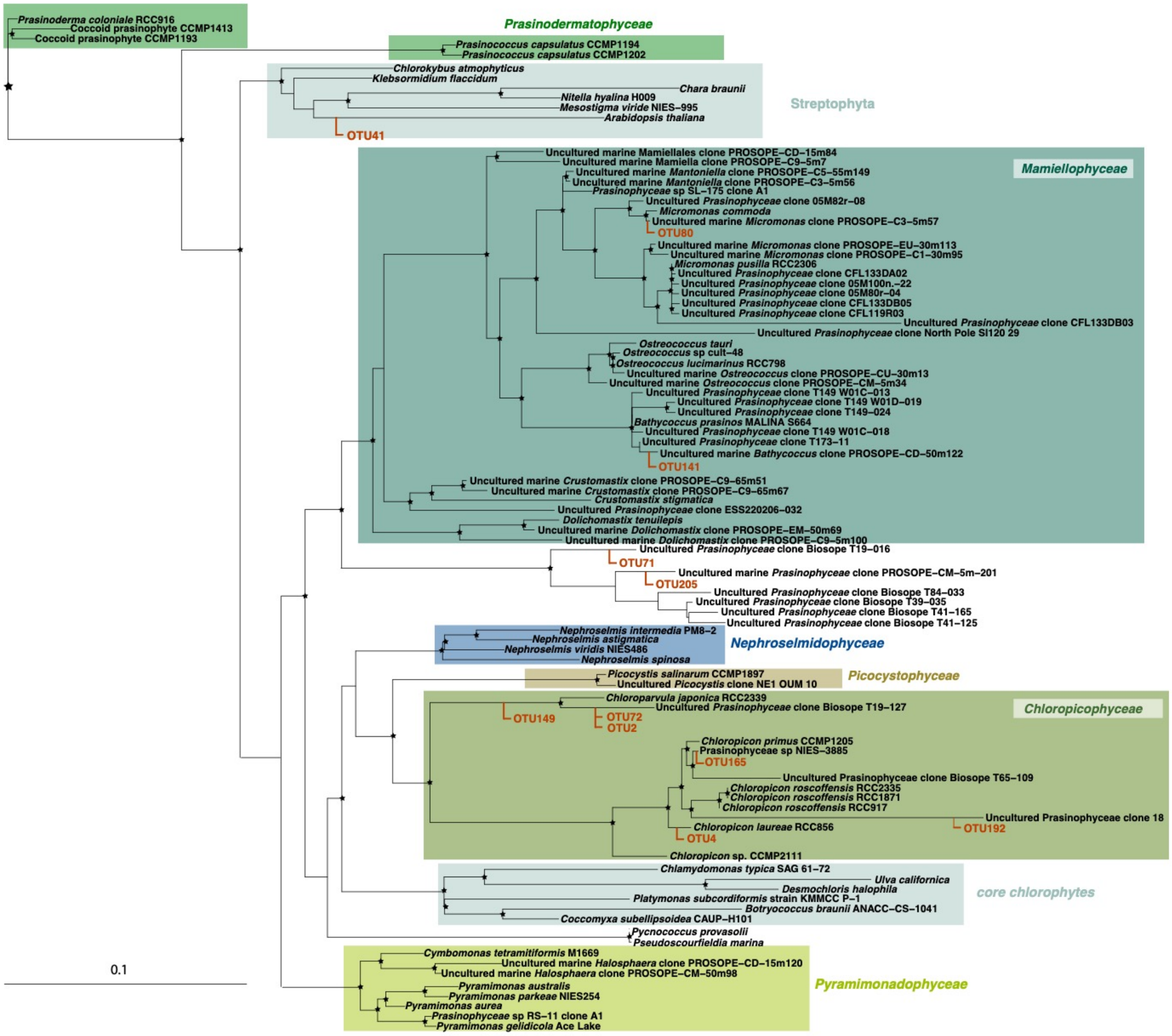

Supplementary Figure S6. Evolutionary placement analysis (EPA) of the Chlorophyta operational taxonomic units (OTUs). The reference tree is rooted *a posteriori* using the Prasinodermophyceae, based on Li et al. (2020). Nodes with bootstrap values above 50% are marked with stars. OTU placements are indicated red branches for recent branching placements. Note that the red branch lengths are not representative of phylogenetic distance.

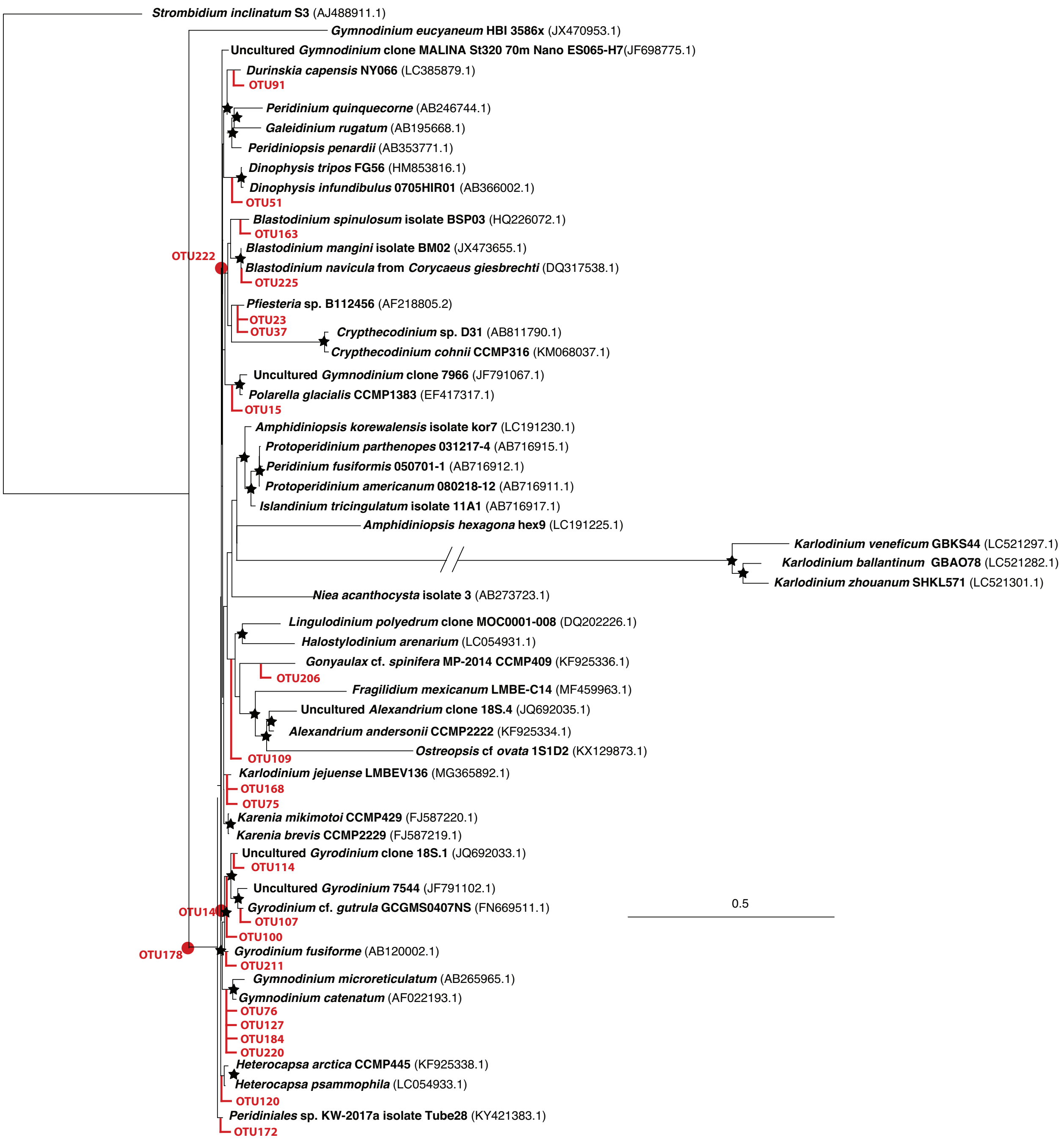

Supplementary Figure S7. Evolutionary placement analysis (EPA) of the dinoflagellate operational taxonomic units (OTUs). The reference tree is rooted with an outgroup from the ciliates, illustrated by *Strombidium inclinatum*. Nodes with bootstrap values above 50% are marked with stars. OTU placements are indicated by red dots for deep branching placements or red branches for recent branching placements. Note that the red branch lengths are not representative of phylogenetic distance.

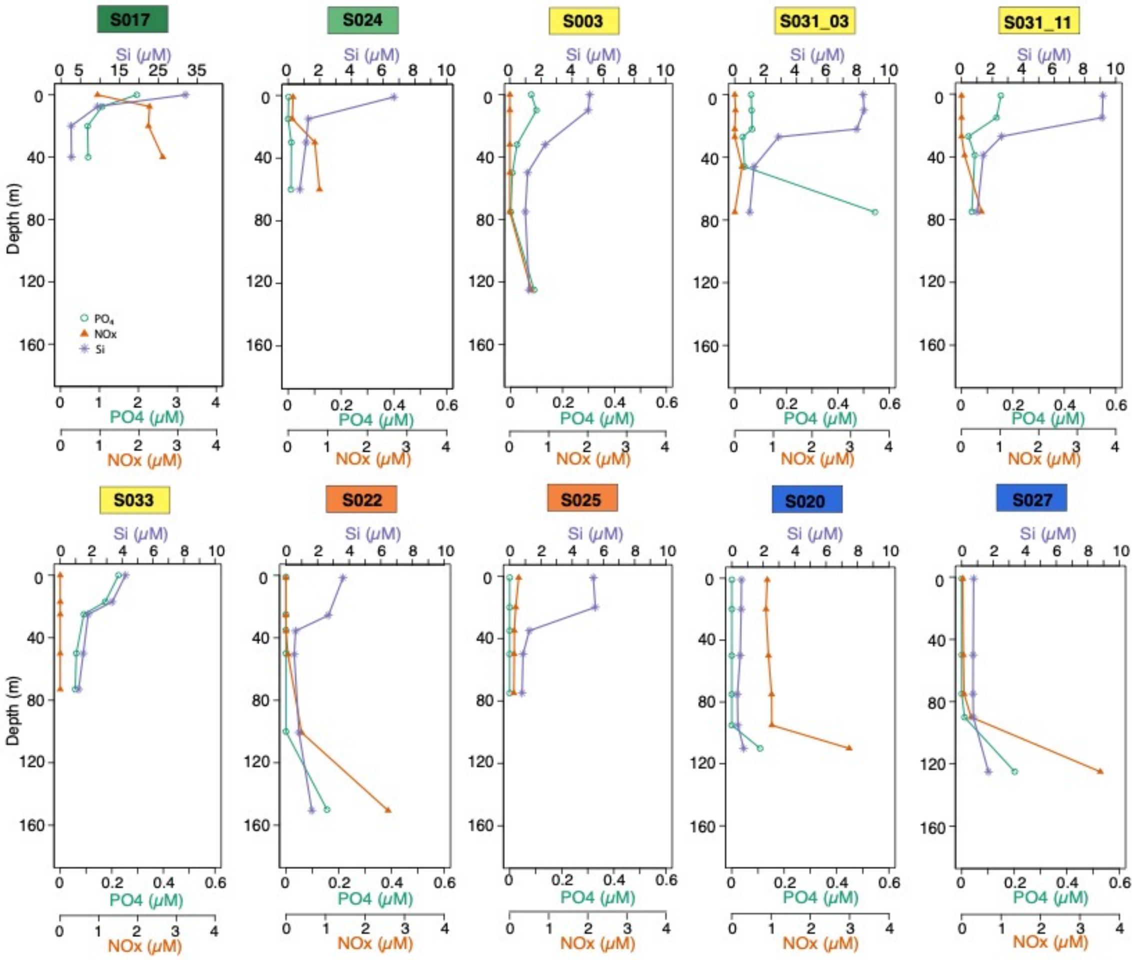

Supplementary Figure S8. Nutrient profiles at each station sampled. PO_4, phosphate; NO_x, nitrate and nitrite; Si, silicate.

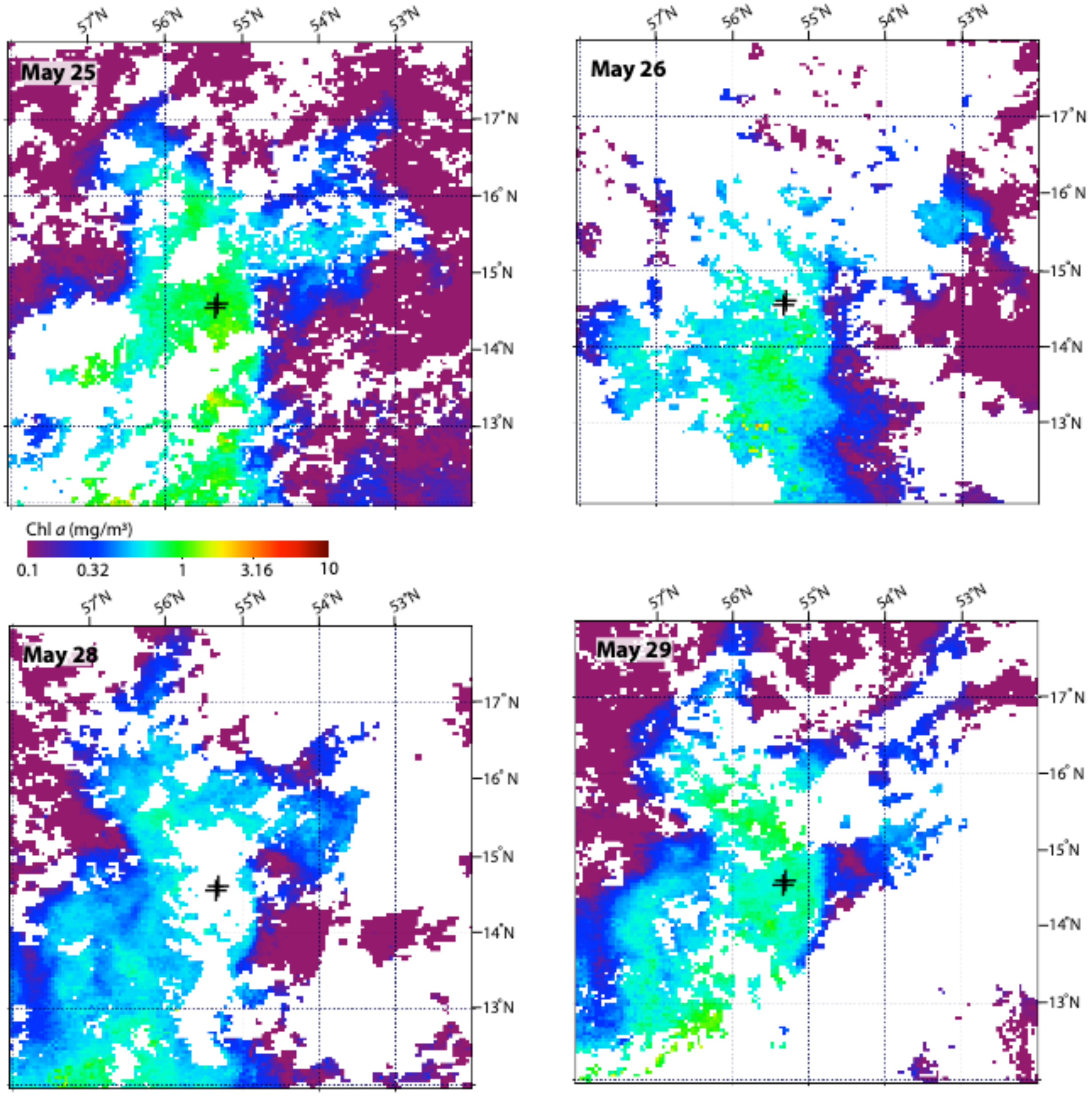

Supplementary Figure S9. Time series of surface chlorophyll distribution at station S031 between May 25th and 29th. Black cross indicates location of S031. White pixels indicate cloud cover (absence of data). The plume, represented by higher chlorophyll concentrations, is shown to be advecting northward through the station, as indicated by the spread of higher chlorophyll concentrations north of 17°N on May 28th and 29th compared to May 25th.
