# Supplementary material for: Small pigmented eukaryote assemblages of the western tropical North Atlantic around the Amazon River plume during spring discharge
Source: Sci Rep. 2021 Aug 10;11:16200. doi: 10.1038/s41598-021-95676-2 (PMC8355221; doi:10.1038/s41598-021-95676-2)
Supplement: Supplementary file 2 — Supplementary Information. [file 41598_2021_95676_MOESM2_ESM.pdf]

Small pigmented eukaryote assemblages of the western tropical North Atlantic around the Amazon River plume during spring discharge.

Sophie Charvet, Eunsoo Kim, Ajit Subramaniam, Joseph Montoya and Solange Duhamel

Supplementary materials

Supp. Mat. SM1: Description of sorted populations

Population P2 was characterized by a wide range of forward scatter (cell size proxy) and red fluorescence (chlorophyll proxy). The P3 population could be distinguished from the *Synechococcus* population as a cluster composed of cells slightly larger and brighter and lacking orange fluorescence (phycoerythrin proxy), while the cells in P4 were slightly larger with brighter red fluorescence. Population P5, composed of cells slightly larger and brighter than the P4 population, was less frequently detected or was present in such low abundances that the population was difficult to separate. The P1 population was only observed in one sample (station S024 at 15m) and consisted of much smaller cells, of a size similar to *Synechococcus*, but with much brighter chlorophyll autofluorescence and absence of phycoerythrin. Similarly, P6 was only observed at station S017 (40m), containing cells bigger than *Synechococcus*, with lower chlorophyll autofluorescence (Fig. 3).

Supp. Mat. SM2: Methodological considerations of FACS sorting.

We encountered some methodological limits, such as a cell density threshold below which the recovery or purity of gated populations was compromised. We observed an abundance threshold below which a small population, such as P4, gating and sorting was imperfect, leading to strong overlaps between gated populations. In addition, our method likely led to the omission of low

abundance populations, such as P1, which could not be gated properly, or because the sorting did not collect enough cells to extract sufficient DNA yields.

Moreover, heterotrophs (cercozoa, fungi and Syndiniales) were inadvertently co-sorted with the pigmented picoeukaryotes. These taxa showed more overlap between populations, probably because they were collected as bycatch and not according to the criteria for PPE sorting. Metazoan sequences in 18S rDNA surveys, excluded from the present data, are generally attributed to cellular contents released from tissue during filtration or to free-floating environmental DNA, which could be retained onto algal cell surfaces, such as the abundant Prasinophyte cells, which sometimes secrete a sticky substance that allows them to form loose colonies or aggregates (Lopes dos Santos, Pollina, et al. 2017).

References

Lopes dos Santos A, Pollina T, Gourvil P, Corre E, Marie D, Garrido JL, Rodríguez F, Noël M-H (2017). Chloropicophyceae, a new class of picophytoplanktonic prasinophytes. *Nature Scientific Reports.***7:**1-20. doi: 10.1038/s41598-017-12412-5

Supp. Mat. SM3: Analyses workflow

```
######## Small pigmented eukaryote assemblages of the western tropical North Atlantic around the Amazon River plume during spring discharge.
### Analyses workflow
## Author: Sophie Charvet (sophie.charvet.1@gmail.com)

########
##### QIIME2

source activate qiime2-2020.2


# importing files from each sequencing run separately:
qiime tools import \
  --type 'SampleData[PairedEndSequencesWithQuality]' \
  --input-path 30-165228665 \
  --input-format CasavaOneEightSingleLanePerSampleDirFmt \
  --output-path 30-165228665.qza

qiime tools import \
  --type 'SampleData[PairedEndSequencesWithQuality]' \
  --input-path 30-170447864 \
  --input-format CasavaOneEightSingleLanePerSampleDirFmt \
  --output-path 30-170447864.qza

qiime tools import \
  --type 'SampleData[PairedEndSequencesWithQuality]' \
  --input-path 30-152766606
  --input-format CasavaOneEightSingleLanePerSampleDirFmt \
  --output-path 30-152766606.qza

# denoising datasets:
qiime dada2 denoise-paired \
  --i-demultiplexed-seqs 30-165228665.qza \
  --p-trim-left-f 15 \
  --p-trim-left-r 15 \
  --p-trunc-len-f 0 \
  --p-trunc-len-r 0 \
  --o-representative-sequences 30-165228665-rep-seqs-dada2.qza \
  --o-table 30-165228665-table-dada2.qza \
  --o-denoising-stats 30-165228665-stats-dada2.qza \
--verbose

qiime dada2 denoise-paired \
  --i-demultiplexed-seqs 30-170447864.qza \
```

```
  --p-trim-left-f 15 \
  --p-trim-left-r 15 \
  --p-trunc-len-f 0 \
  --p-trunc-len-r 0 \
  --o-representative-sequences 30-170447864-rep-seqs-dada2.qza \
  --o-table 30-170447864-table-dada2.qza \
  --o-denoising-stats 30-170447864-stats-dada2.qza \
--verbose

qiime dada2 denoise-paired \
  --i-demultiplexed-seqs 30-152766606.qza \
  --p-trim-left-f 15 \
  --p-trim-left-r 15 \
  --p-trunc-len-f 0 \
  --p-trunc-len-r 0 \
  --o-representative-sequences 30-152766606-rep-seqs-dada2.qza \
  --o-table 30-152766606-table-dada2.qza \
  --o-denoising-stats 30-152766606-stats-dada2.qza \
--verbose

# Merging the feature tables and feature data from all three runs:
qiime feature-table merge \
  --i-tables 30-152766606-table-dada2.qza \
  --i-tables 30-165228665-table-dada2.qza \
  --i-tables 30-170447864-table-dada2.qza \
  --o-merged-table merged_feature_table.qza

qiime feature-table merge-seqs \
  --i-data 30-152766606-rep-seqs-dada2.qza \
  --i-data 30-165228665-rep-seqs-dada2.qza \
  --i-data 30-170447864-rep-seqs-dada2.qza \
  --o-merged-data merged_repseqs.qza

# Clustering:
qiime vsearch cluster-features-closed-reference \
  --i-table merged_feature_table.qza \
  --i-sequences merged_repseqs.qza \
  --i-reference-sequences SILVA_97_otus_18S.qza \
  --p-perc-identity 0.97 \
  --o-clustered-table merged_table-cr-97.qza \
  --o-clustered-sequences merged_repseqs-cr-97.qza \
  --o-unmatched-sequences merged_unmatched-cr-97.qza

# Chimera filtering:
qiime vsearch uchime-denovo \
--i-table merged_table-cr-97.qza \
```

```
--i-sequences  merged_repseqs-cr-97.qza \
--output-dir  merged_uchime-cr

# Filter out all features corresponding to chimeras (in the FeatureTable[Frequency]):
qiime feature-table filter-features \
   --i-table merged_table-cr-97.qza \
   --m-metadata-file merged_uchime-cr/nonchimeras.qza \
   --o-filtered-table merged_table-cr-97-nonchim.qza

qiime feature-table filter-seqs \
   --i-data  merged_repseqs-cr-97.qza \
   --m-metadata-file merged_uchime-cr/nonchimeras.qza \
   --o-filtered-data merged_repseqs-cr-97-nonchim.qza

# Remove singletons:
qiime feature-table filter-features \
   --i-table merged_table-cr-97-nonchim.qza \
   --p-min-samples 2 \
   --o-filtered-table merged_table-cr-97-abundances.qza

qiime feature-table filter-seqs \
   --i-data  merged_repseqs-cr-97-filtchim.qza \
   --i-table merged_table-cr-97-abundances.qza \
   --o-filtered-data merged_repseqs-cr-97-abundances.qza

# Rarefication (subsampling) to equalize #reads per sample:
qiime feature-table rarefy \
    --i-table merged_table-cr-97-abundances.qza \
    --p-sampling-depth 2000 \
    --o-rarefied-table merged_table-cr-97-abundRarefied2000.qza

qiime feature-table filter-seqs \
    --i-data merged_repseqs-cr-97-abundances.qza \
    --i-table merged_table-cr-97-abundRarefied2000.qza \
    --o-filtered-data merged_repseqs-cr-97-abundRarefied2000.qza

# Exporting OTU table for further analyses in R:
qiime tools export \
    --input-path merged_table-cr-97-abundRarefied2000.qza \
    --output-path merged_table-cr-97-abundRarefied2000_OTUbiom

biom convert -i merged_table-cr-97-abundRarefied2000_OTUbiom/feature-table.biom -
o merged_table-cr-97-abundRarefied2000_OTUbiom.txt --to-tsv

# Taxonomic classification:
## importing reference PR2 database:
```

```
qiime tools import \
  --type 'FeatureData[Sequence]' \
  --input-path pr2_version_4.14.0_SSU_mothur.fasta \
  --output-path pr2_version_4.14.0_SSU_mothur.fasta.qza

## importing reference PR2 taxonomy:
qiime tools import \
  --type 'FeatureData[Taxonomy]' \
  --input-path pr2_version_4.14.0_SSU_mothur.tax \
  --input-format HeaderlessTSVTaxonomyFormat \
  --output-path pr2_version_4.14.0_SSU_mothur.tax.qza

## Trimming reference database:
qiime feature-classifier extract-reads \
--i-sequences pr2_version_4.14.0_SSU_mothur.fasta.qza \
--p-f-primer CCGCGGTAATTCCAGCTC \
--p-r-primer GATCCCYYAACTTTCGTTCTTGA \
--o-reads pr2_version_4.14.0_SSU_mothur.fasta.trim.qza \
--verbose

## training the classifier:
qiime feature-classifier fit-classifier-naive-bayes \
--i-reference-reads pr2_version_4.14.0_SSU_mothur.fasta.trim.qza \
--i-reference-taxonomy pr2_version_4.14.0_SSU_mothur.tax.qza \
--o-classifier pr2_version_4.14.0_classifier_18S \
--verbose

## OTU classification:
qiime feature-classifier classify-sklearn \
  --i-classifier pr2_version_4.14.0_classifier_18S.qza \
  --i-reads axo2/merged_repseqs2-cr-97-abundRarefied2000.qza \
  --o-classification Taxo2/merged_Taxo2-cr-97-abundRarefied2000_pr2_sklearn.qza \
  --verbose

qiime taxa barplot \
  --i-table merged_table-cr-97-abundRarefied2000.qza \
  --i-taxonomy merged_Taxo-cr-97-abundRarefied2000_pr2_sklearn.qza \
  --m-metadata-file EN614-metadataTaxClass.txt \
  --o-visualization merged_Taxo-cr-97-abundRarefied2000_pr2_sklearnViz \
  --verbose

## Export taxonomy abundance table
qiime tools export \
    --input-path merged_Taxo-cr-97-abundRarefied2000_pr2_sklearnViz.qzv \
    --output-path merged_Taxo-cr-97-abundRarefied2000_pr2_sklearnViz
```

```
## Export taxonomy of each OTU
qiime tools export \
    --input-path merged_Taxo-cr-97-abundRarefied2000_pr2_sklearn.qza \
    --output-path merged_Taxo-cr-97-abundRarefied2000_pr2_sklearn-OTUtax

# Beta-diversity (UniFrac distance, UPGMA):
qiime phylogeny align-to-tree-mafft-fasttree \
--i-sequences merged_repseqs-cr-97-abundRarefied2000.qza \
--o-alignment merged_repseqs-cr-97-abundRarefied2000MAFFTalign.qza \
--o-tree merged_repseqs-cr-97-abundRarefied2000MAFFTTree.qza \
--o-masked-alignment merged_repseqs-cr-97-abundRarefied2000MAFFTalign-masked.qza \
--o-rooted-tree merged_repseqs-cr-97-abundRarefied2000MAFFTTreeRooted.qza \
--verbose

qiime diversity beta-phylogenetic \
--i-table merged_table-cr-97-abundRarefied2000.qza \
--i-phylogeny merged_repseqs-cr-97-abundRarefied2000MAFFTTreeRooted.qza \
--p-metric weighted_unifrac \
--o-distance-matrix merged_repseqs-cr-97-abundRarefied2000wUnifrac.qza \
--verbose

qiime diversity beta-rarefaction \
--i-table merged_table-cr-97-abundRarefied2000.qza \
--p-metric weighted_unifrac \
--p-clustering-method upgma \
--m-metadata-file EN614-EnvData.txt \
--p-sampling-depth 1000 \
--i-phylogeny merged_repseqs-cr-97-abundRarefied2000MAFFTTreeRooted.qza \
--p-correlation-method spearman \
--o-visualization merged_repseqs-cr-97-abundRarefied2000wUnifracUPGMA \
--verbose

qiime tools export \
    --input-path merged_repseqs-cr-97-abundRarefied2000wUnifracUPGMA.qzv \
    --output-path merged_repseqs-cr-97-abundRarefied2000wUnifracUPGMA

# ANCOM analyses (on groups of samples)
(Example here of P3 populations)
qiime feature-table filter-samples \
  --i-table merged_table-cr-97-abundRarefied2000.qza \
  --m-metadata-file EN614-metadataTaxClass.txt \
  --p-where "Pop_descr='P3-PE'" \
  --o-filtered-table P3-merged_table-cr-97-abundRarefied2000.qza

qiime composition add-pseudocount \
  --i-table P3-merged_table-cr-97-abundRarefied2000.qza \
```

```
  --o-composition-table P3-merged_table-cr-97-abundRarefied2000-comp.qza

qiime composition ancom \
  --i-table P3-merged_table-cr-97-abundRarefied2000-comp.qza \
  --m-metadata-file EN614-metadataTaxClass2.txt \
  --m-metadata-column Station_type \
  --o-visualization P3-ancom-Station_type.qzv

###########
##### Evolutionary placement analyses (EPA)
(Example here of Bacillariophyceae)

qiime tools export \
    --input-path merged_Taxo2-cr-97-abundRarefied2000.qza \
    --output-path for_EPA/

qiime tools export \
    --input-path merged_repseqs-cr-97-abundRarefied2000.qza \
    --output-path for_EPA/

## Extract list of Bacillariophyceae OTUs (13 in total):
grep "Bacillariophyceae" for_EPA/taxonomy.tsv | awk '{print $1}' >
Bacillariophyceae_OTUs.txt

## extract representative sequences corresponding to the Bacillario OTUs:
grep -A 1 -f  Bacillariophyceae_OTUs.txt for_EPA/dna-sequences.fasta >
Bacillariophyceae_Query.fas

## Make reference alignment with fasta of reference Bacillariophyceae sequences from NCBI:
MUSCLE\ v3.8.31/muscle3.8.31_i86darwin32 -in Bacillariophyceae_RefRooted.fas -out
Bacillariophyceae_RefRootedAligned.fa

## transform alignment to .phy format (with AliView)

## Phylogenetic reconstruction (reference tree):
./raxmlHPC -s Bacillariophyceae_RefRootedAligned.phy -m GTRGAMMA -# 10 -b 100 -o
rKY464903.1 -n Bacillariophyceae_RefRootedTree

## Add bootstraps to ref tree:
./raxmlHPC -f b -m GTRCAT -s Bacillariophyceae_RefRootedAligned.phy -z
RAxML_bootstrap.Bacillariophyceae_RefRooted -t
RAxML_bestTree.Bacillariophyceae_RefRooted -o rKY464903.1 -
n Bacillariophyceae_RefRooted_BStree

## Make alignment with reference and query sequences:
```

MUSCLE\ v3.8.31/muscle3.8.31_i86darwin32 -in Bacillariophyceae_RefQueryRooted.fas -out Bacillariophyceae_RefQueryRootedAligned.fas

transform alignment to .phy format (with AliView)

Placement of query reads:
./raxmlHPC -f v -s Bacillariophyceae_RefQueryRootedAligned.phy -m GTRGAMMA -t RAxML_bestTree.Bacillariophyceae_RefRooted -n Bacillariophyceae_EPArootedTree
