# Supplementary material for: Small pigmented eukaryote assemblages of the western tropical North Atlantic around the Amazon River plume during spring discharge
Source: Sci Rep. 2021 Aug 10;11:16200. doi: 10.1038/s41598-021-95676-2 (PMC8355221; doi:10.1038/s41598-021-95676-2)
Supplement: Supplementary file 3 — Supplementary Table S1. [file 41598_2021_95676_MOESM3_ESM.pdf]

Supp. Table S1: Total small phytoplankton (≤ 5μm) cell concentrations (cells/mL), and the respective proportions of picocyanobacteria and small pigmented eukaryotes.

Station	Depth	Total cells ≤5μm	% Prochlorococcus	% Synechococcus	%SPE
S003	surface	91059	1.88	96.53	1.59
S017	deep	8231	71.04	10.17	18.79
S020	surface	228100	92.61	6.33	1.06
S022	surface	252183	65.93	32.82	1.25
S022	subsurf	264636	66.17	32.86	0.97
S022	CM	116195	66.90	27.35	5.75
S024	surface	152702	7.94	90.11	1.95
S024	CM	32436	7.96	85.66	6.38
S024	below-CM	30258	9.66	77.59	12.76
S024	deep	14146	85.90	6.25	7.85
S025	surface	165599	41.53	56.69	1.77
S025	CM	81262	52.33	45.16	2.51
S027	surface	255852	97.49	1.99	0.52
S027	CM	61263	90.30	4.08	5.62
S031_03	surface	180507	22.13	76.75	1.12
S031_03	CM	238359	71.47	27.43	1.10
S031_11	surface	223696	24.15	74.32	1.54
S031_11	CM	311177	76.09	23.34	0.57
