# Supplementary material for: Small pigmented eukaryote assemblages of the western tropical North Atlantic around the Amazon River plume during spring discharge
Source: Sci Rep. 2021 Aug 10;11:16200. doi: 10.1038/s41598-021-95676-2 (PMC8355221; doi:10.1038/s41598-021-95676-2)
Supplement: Supplementary file 4 — Supplementary Table S2. [file 41598_2021_95676_MOESM4_ESM.pdf]

Supp. Table S2: Biomass estimates of picocyanobacteria and small pigmented picoeukaryotes. Estimates are based on biovolumes converted to biomass by an average conversion coefficient used by Worden et al., 2004. Biomass is in µg C m-3. CM, chlorophyll maximum

**Station**	**Depth**	Prochlorococcus	Synechococcus	SPE
S003	surface	125.15	12564.81	11792.10
S017	deep	427.93	119.59	8228.81
S020	surface	15460.95	2063.15	34319.85
S022	surface	12168.25	11832.06	33149.01
S022	subsurf	12814.94	12430.25	36488.56
S022	CM	5688.97	4542.79	26984.00
S024	surface	887.86	19668.13	34019.32
S024	CM	188.93	3971.79	22917.77
S024	below-CM	213.81	3355.68	14668.12
S024	deep	889.30	126.48	5936.28
S025	surface	5033.88	13420.10	44484.38
S025	CM	3111.97	5246.21	11443.36
S027	surface	18255.44	726.47	23352.51
S027	CM	4048.78	357.16	83284.61
S031_03	surface	2922.96	19803.63	20159.40
S031_03	CM	12467.09	9345.72	50098.01
S031_11	surface	3952.95	23764.14	31689.69
S031_11	CM	17328.78	10380.87	32030.92
